# Structural Analysis of Hyaluronic Acid Fillers Using Nuclear Magnetic Resonance: Implications for Quality Control and Clinical Performance

**DOI:** 10.3390/polym16202878

**Published:** 2024-10-12

**Authors:** Won Lee, Eun-Jung Yang

**Affiliations:** 1 Yonsei E1 Plastic Surgery Clinic, Anyang 14072, Republic of Korea; e1clinic@hanmail.com; 2 Department of Plastic and Reconstructive Surgery, Institute of Human Tissue Regeneration, Yonsei University College of Medicine, Seoul 03721, Republic of Korea

**Keywords:** hyaluronic acid, hyaluronic acid filler, 1,4-butanediol diglycidyl ether (BDDE), nuclear magnetic resonance (NMR)

## Abstract

Potential disruptions in the biocompatibility of hyaluronic acid (HA) fillers can arise with mono-linked 1,4-butanediol diglycidyl ether (BDDE) or unreacted (pendant) 1,4-butanediol di-(propan-2,3-diolyl) ether. Assessing the filler’s degree of modification involves evaluating improperly cross-linked BDDE. This study analyzed commercially available HA fillers using nuclear magnetic resonance (NMR), focusing on key parameters, such as the degree of modification (MoD), the cross-linker ratio (CrR), and the degree of cross-linking. We assessed thirteen commercially available HA fillers using NMR. The samples were placed in an NMR instrument, and each sample was analyzed for 26 h, including MoD and CrR assessments. MoD ^1^H ranged from 17.065% to 2.239%, MoD ^13^C ranged from 12.567% to 1.947%, and CrR ^13^C ranged from 0.394 to 0.014. Significant distinctions were observed in the CrR ^13^C values when the MoD values of the products were similar. This study underscores the importance of considering the MoD and the CrR together to ensure optimal cross-linking and minimize the risks associated with residual BDDE impurities. Utilizing NMR for HA gel characterization provides valuable insights regarding product quality control, safety assessments, and clinical performance evaluations for esthetic interventions, contributing to filler product improvements. Further studies correlating NMR findings with real-world outcomes are essential for ensuring safety and efficacy.

## 1. Introduction

**Figure 1:**
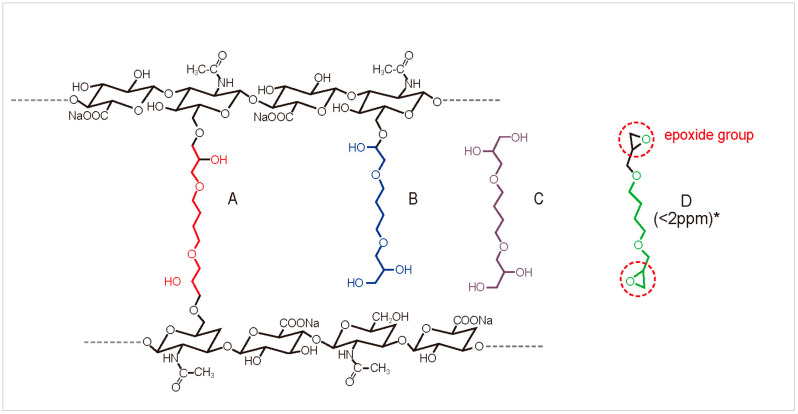
Different types of 1,4-butanediol diglycidyl ether: (**A**) double-cross-linked; (**B**) mono-linked or unreacted (pendant); (**C**) inactivated; (**D**) native. * 2 ppm or lower is generally accepted as a safe threshold for the residual amount of BDDE.

Dermal filler injection is one of the most frequently performed esthetic procedures [1]. Hyaluronic acid (HA) fillers, in particular, are the most commonly used because HA is a naturally occurring substance in the skin, making it highly biocompatible [2]. However, natural HA is rapidly broken down by the body, typically within 1–2 days. HA molecules are chemically cross-linked to extend their presence and effects on the skin, creating a more stable and durable structure [3]. This cross-linking process transforms HA into a gel-like substance that resists degradation, allowing the filler’s effects to last from several months to over a year [4,5]. The degree and type of cross-linking directly influence the filler’s properties, such as viscoelasticity, spreadability, and longevity, making it suitable for various cosmetic applications. However, it is important to note that not all cross-links formed by BDDE (1,4-butanediol diglycidyl ether) between two HA chains are optimal [6]. When BDDE reacts with HA to form bonds, it can result in four different forms, not all of which involve true cross-linking. Among these, only the form that properly cross-links with HA is considered valid, while the others are regarded as impurities. A previous study identified four distinct forms of BDDE interaction with HA: double- or mono-linked 1,4-butanediol di-(propan-2,3-diolyl) ether (BDPE) residues, inactivated forms, and native HA (Figure 1) [6].

Concerns regarding the biocompatibility of HA fillers arise with mono-linked BDPE or unreacted (pendant) BDDE, as the presence of unreacted BDDE may disrupt the natural compatibility of HA fillers with body tissues, thereby affecting the integration of fillers into the injection site. Improper cross-linking fails to contribute to the elasticity of the filler product [7], and mono-linked BDDE can be considered impurities [8]. The HA filler’s degree of modification (MoD) should be calculated to ascertain the extent of improperly cross-linked BDDE. Residual BDDE has the potential to trigger inflammatory reactions in the surrounding tissues, which can manifest as redness, swelling, and discomfort [9]. The severity and duration of the inflammatory reaction can vary depending on several factors, such as the amount of residual BDDE, an individual’s immune response, and the specific filler’s characteristics [10]. Excessive or prolonged inflammation may contribute to patient dissatisfaction, necessitating corrective measures. Consequently, HA filler products should ideally contain minimal improperly cross-linked BDDE. Despite being a critical issue potentially associated with delayed immune responses, analyses of unreacted BDDE in commercially available HA fillers have been limited.

This study aimed to analyze the variety of hyaluronic acid fillers available in the market and address their structural characteristics. Unlike previous studies that primarily focused on lab-made cross-linked HA, our research investigated 13 commercially available fillers using ^1^H and ^13^C NMR spectroscopy. A detailed analysis of the modification degree (MoD), as well as the cross-linking ratio (CrR) and degree of crosslinking (CrD), was performed using ^13^C NMR, providing a visual representation of how hyaluronic acid and BDDE are structurally combined.

The novelty of this research lies in its comprehensive approach to comparing a wide range of commercial fillers and elucidating their structural characteristics through advanced NMR techniques. Additionally, we discussed the clinical implications of our findings, emphasizing the importance of understanding the structural differences in fillers to mitigate potential adverse effects.

## 2. Materials and Methods

### 2.1. HA Fillers

Thirteen commercially available HA fillers were assessed, including Restylane Volyme (lot 17031-1; Galderma, Uppsala, Sweden), Restylane Defyne (lot 18681-1; Galderma), Restylane Lyft (lot 19765; Galderma), Juvederm Voluma (lot 1000561748; Allergan, Irvine, CA, USA), Belotero Volume (lot B00016110; Merz Pharmaceuticals, Frankfurt, Germany), Teosyal RHA4 ( lot 22372DL03128; TEOXANE Laboratories, Geneva, Switzerland), Lorient No. 6 (lot J21001; Joonghun Pharmaceutical, Seoul, Republic of Korea), Neuramis Volume (lot C523010A; Medy-Tox, Seoul, Republic of Korea), The Chaeum Premium No. 4 (lot BLD21008; HUGEL, Seoul, Republic of Korea), Elravie Deep (lot D6016002AA; Humedix, Seoul, Republic of Korea), Eptq S500 (lot YLC22006; Jetema, Seoul, Republic of Korea), QTfill SubQ (lot QPAI21001G; S.THEPHARM, Seoul, Republic of Korea), and Youthfill Shape (lot YDE19013; RFBio, Seoul, Republic of Korea).

### 2.2. Sample Preparation

**Figure 2:**
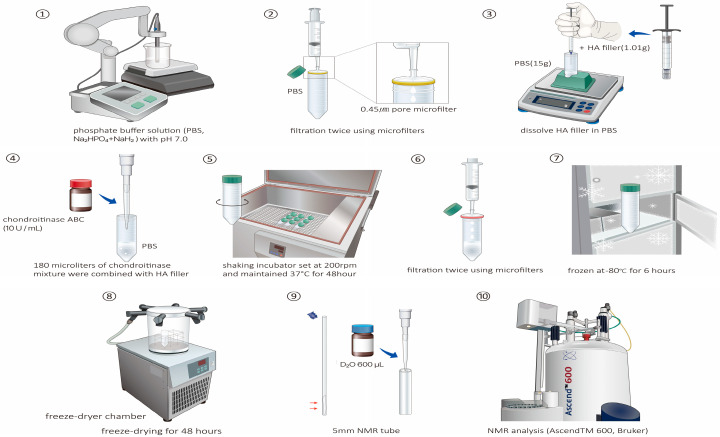
Schematic representation of the sample preparation process for nuclear magnetic resonance (NMR) analysis of the hyaluronic acid (HA) filler sample, which includes the preparation of phosphate-buffered solution (pH 7.0), enzymatic treatment with chondroitinase ABC, incubation in a shaking chamber, filtration through microfilters, and freeze-drying for preservation. For filtration and preservation, the solution is filtered twice using microfilters of different pore sizes (0.45, 0.2, and 0.1 µm) after incubation. The filtered solution is then frozen at −80 °C for 6 h and sealed according to the NMR analysis setup, and the parameters for the HA filler sample are applied. The freeze-dried sample is reconstituted in 600 µL of D_2_O solution in a 5 mm NMR tube. The tube is then inserted into an NMR instrument (Ascend™ 600, Bruker) for analysis; the specific parameters used for the ^1^H-NMR (32 scans; D1 time, 25 s) and ^13^C-NMR (4096 scans; D1 time, 15 s) experiments are provided. The paraffin is subjected to freeze-drying for 48 h to ensure sample integrity.

The preparation of the HA fillers for NMR analysis is summarized in Figure 2. A phosphate-buffered solution (Na_2_HPO_4_ + NaH_2_, 1 mM, pH of 70) was prepared, and a pH analyzer (S500_Basic; Mettler Toledo, Columbus, OH, USA) was used to ensure an accurate pH of 7.0. The PBS was then passed through a 0.45 μm microfilter to remove any particulate matter or impurities that could interfere with the NMR analysis. Then, 1.01 g HA filler was dissolved in 15 g of PBS to ensure that the HA filler was adequately dissolved in the buffer, facilitating subsequent enzymatic treatment. Subsequently, 10 U chondroitinase ABC (Sigma-Aldrich, St. Louis, MO, USA) was dissolved in 1 mL of PBS. Then, 180 μL of chondroitinase mixture was combined with the HA filler in a cold environment. Chondroitinase is an enzyme used for the structural analysis of HA fillers because it selectively degrades chondroitin sulfate, which is often present as a contaminant in commercially available HA fillers and can interfere with the accurate structural analysis of HA fillers. The solution was transferred to a shaking incubator chamber (DA-SI-LL; Dong-A Science, Seoul, Republic of Korea) set at 200 rpm and maintained at 37 °C for 48 h. This prolonged incubation period ensured the complete dissolution of HA by chondroitinase, which may provide insights into its structural characteristics. The solution was then passed twice through a microfilter (Minisart^®^ syringe filters; S6555 [0.45 μm], S6534 [0.2 μm], and 16,553 [0.1 μm]) to remove any remaining particulates or undissolved components. Subsequently, the solution was frozen at −80 °C for 6 h and sealed with paraffin. The sealed solution was freeze-dried for 48 h using a freeze-dryer chamber (Operon Advantech Co., Gimpo-si, Gyeonggi-do, Republic of Korea) to preserve the sample’s integrity.

### 2.3. NMR Analysis

The freeze-dried sample was inserted into a 5 mm NMR tube, to which 600 µL D_2_O solution was added, and the tube was sealed. The sealed tube was placed in an NMR instrument (Ascend™ 600; Bruker, Billerica, MA, USA), and each sample was analyzed for 26 h. For ^1^H-NMR, 32 scans and a recycle delay (D1) of 25 s were used. For ^13^C-NMR, 4096 scans and a D1 time of 15 s were used.

### 2.4. HA Gel Characterization

We used the following terms proposed by Kenne et al. [11] to characterize HA hydrogels cross-linked with BDDE:

- Degree of modification (MoD): This measures the stoichiometric ratio of the sum of the mono- and double-linked 1,4-butanediol di-(propan-2,3-diolyl) ether (BDPE) residues and the HA disaccharide units. A higher MoD percentage indicates more cross-linking modifications than the acetyl group. Essentially, the MoD signifies how HA deviates from its natural state, potentially acting as a foreign substance in the body.
- Cross-linking ratio (CrR): This denotes the fraction of the double-linked cross-linker residues compared with all linked cross-linkers and represents a measure of cross-linking efficiency. A higher CrR indicates a safer and more effective filler, as it contains fewer foreign substances and is efficiently cross-linked.
- Degree of cross-linking (CrD): This ratio reflects the stoichiometric relationship between the double-linked BDPE residues and the HA disaccharide units. A high CrD suggests structural stability and longevity if the HA filler is appropriately cross-linked. However, a high CrD value does not always equate to a safe and effective filler. Structural stability is apparent when both the CrR and the CrD are high. Nonetheless, even under such circumstances, a high MoD increases the likelihood of the filler being perceived as a foreign substance.

When analyzing HA hydrogels, ^1^H-NMR has been used to determine the MoD [12], whereas ^13^C-NMR has been used to determine the CrD [13]. In the present study, the CrD values of the selected HA fillers were determined using the NMR-based approach described by Wende et al. [11]. The three primary parameters were then calculated using the following formulas:

MoD ^1^H (%) = (Iδ^H1.7^/4)/(Iδ^H2.1^/3) × 100, (1)

MoD ^13^C (%) = (Iδ^c25.2^/2)/Iδ^c1.9−22.6^ × 100, (2)

CrR = 1 − Iδ^C62.7^/(Iδ^c25.2^/2), (3)

CrD (%) = (CrR × MoD) × 100.(4)

## 3. Results

### 3.1. Structural Analysis Using NMR

Table 1 presents the various HA fillers based on the key cross-linking parameters, including the MoD (^1^H and ^13^C), the CrR (^13^C), and the CrD (^13^C). A significant range was observed for both MoD ^1^H and MoD ^13^C across the tested fillers. Specifically, MoD ^1^H ranged from 17.065% to 2.239%, whereas MoD ^13^C ranged from 12.567% to 1.947%. Despite these absolute differences, the relative levels remained comparatively consistent. 

### 3.2. Characteristics Affecting the Biocompatibility and Performance

**Figure 3:**
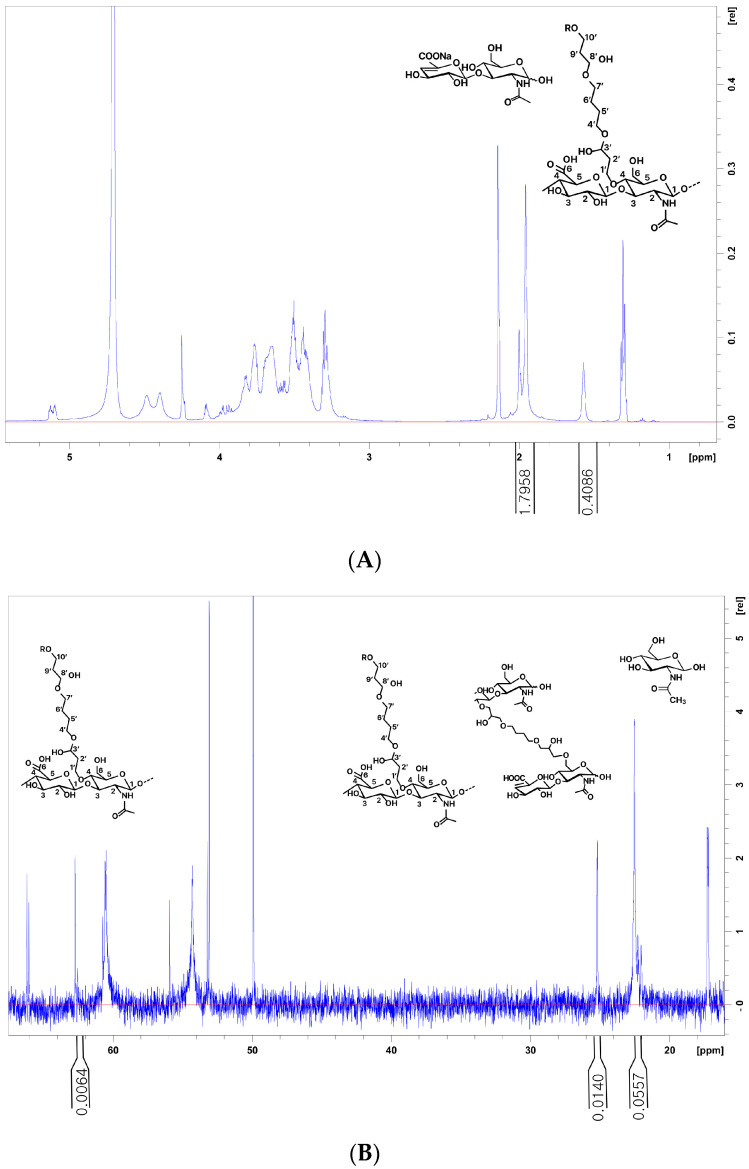
Different spectra of ^1^H and ^13^C nuclear magnetic resonance (NMR) analysis of the hyaluronic acid (HA) filler samples: Product 5 has a high MoD but a relatively low CrR value of 0.086 among the tested fillers. (**A**) The N-acetyl signal (CH_3_) from the HA and BDPE signals (H5′, H6′) used for the determination of the MOD. MoD ^1^H (%) = (Iδ^H1.7^/4)/(Iδ^H2.1^/3) × 100. (**B**) The C10′ signals on mono-linked BDPE are the C5′ and C6′ of both mono- and cross-linked BDPE used to determine CrR, CrR = 1 − I^δC62.7^/(I^δC25.2^/2). The signals and CH_3_ of N-Acetyl glucosamine are used to determine the MoD, MoD (%) = (I^δC25.2^/2)/I^δC21.9−22.6^ × 100.

**Figure 4:**
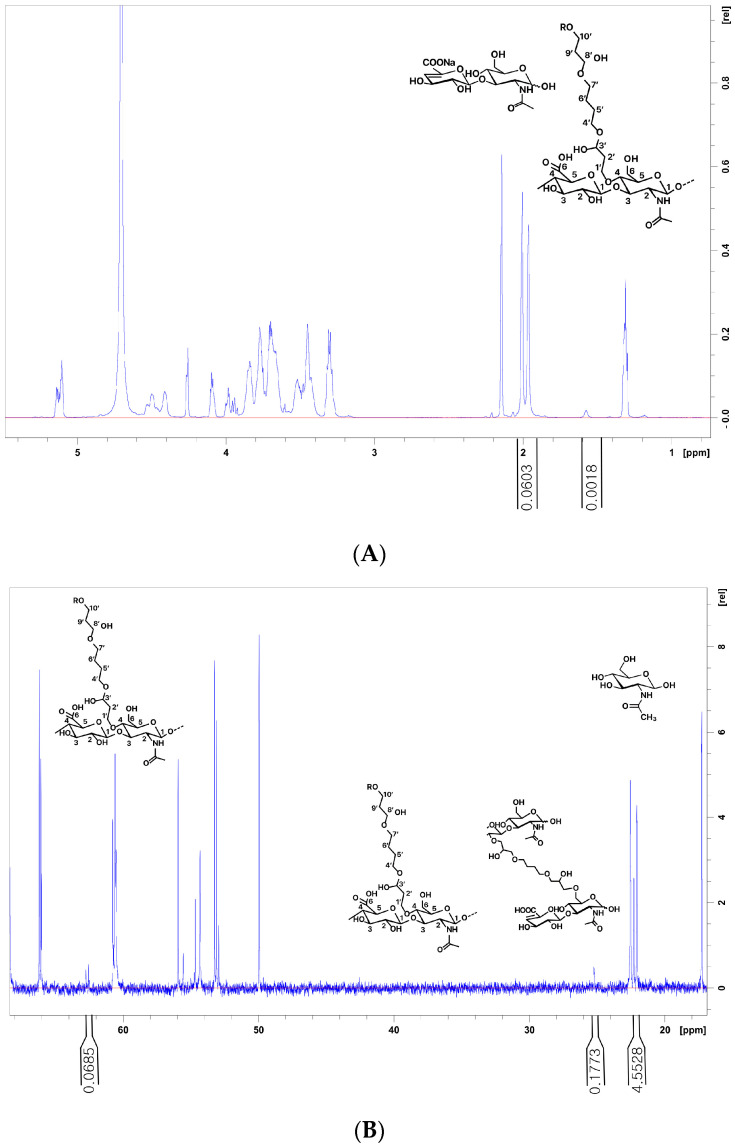
Different spectra of ^1^H and ^13^C nuclear magnetic resonance (NMR) analysis of the hyaluronic acid (HA) filler samples: (**A**,**B**) Product 7 has a low MoD but a high CrR value of 0.222 among the tested fillers. (**A**) The N-acetyl signal (CH_3_) from HA and BDPE signals (H5′, H6′) used for the determination of the MOD. MoD ^1^H (%) = (Iδ^H1.7^/4)/(Iδ^H2.1^/3) × 100. (**B**) The C10′ signals on mono-linked BDPE are the C5′ and C6′ of both mono- and cross-linked BDPE used to determine the CrR, CrR = 1 − I^δC62.7^/(I^δC25.2^/2). The signals and CH_3_ of N-Acetyl glucosamine are used to determine the MoD, MoD (%) = (I^δC25.2^/2)/I^δC21.9−22.6^ 100. MoD, degree of modification; CrR, cross-linking ratio.

Products 5 and 9 showed the highest MoD ^13^C values of 12.567% and 12.101%, respectively, indicating an extensive modification (Figure 3 and Figure 4). Interestingly, although both products exhibited high MoD values, their CrD values diverged significantly. Specifically, product 9 exhibited a prominent CrD of 4.034%, whereas product 5 exhibited a considerably lower value of 1.077%. Product 8, similar to product 5, had a high MoD among the tested fillers; however, its cross-linking efficiency was as low as 0.014%, suggesting that it may contain high amounts of unreacted BDDE.

**Figure 5:**
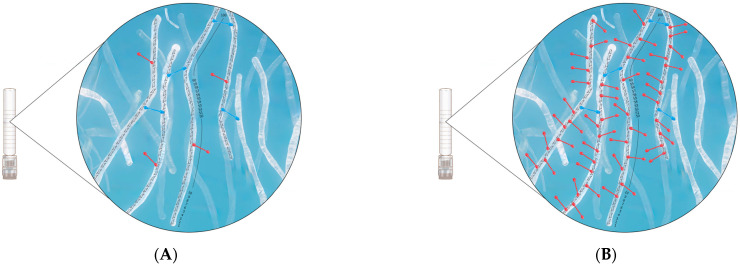
Schematic of the mono- and double-linked 1,4-butanediol di-(propan-2,3-diolyl) ether (BDPE) residues and hyaluronic acid (HA) disaccharide units. The higher MoD percentage indicates more cross-linking modifications. Although a higher MoD is essential, it is equally important to consider the degree of properly (blue-colored) or improperly (red-colored) attached BDDE in conjunction with CrR rather than MoD alone. Instances where MoD is high, but CrR is low indicate inadequate cross-linking, which leads to increased unreacted BDDE (mono-linked BDPE residues) and reduced G′ and cohesiveness: (**A**) Product 5 exhibits a high MoD, but its CrR value is relatively low at 0.086; (**B**) Product 7 has a low MoD but a high CrR value of 0.222. MoD, degree of modification; CrR, cross-linking ratio.

While product 5 exhibited a high MoD, its CrR value was relatively low (0.086) among the tested fillers. Conversely, product 7 had a low MoD, yet its CrR value was high (0.222) among the tested fillers (Figure 5). The CrR value of product 9 was 0.333, closely mirroring that of product 13 (0.343). However, a notable distinction arose when examining the MoD values, with product 9 exhibiting nearly double the MoD of product 13. This disparity potentially indicates a higher absolute pendant BDDE residue value for product 9.

Interestingly, product 2, despite having a high MoD value of 9.723%, had a low CrD 13C value (0.824%) among the assessed fillers. This observation suggested that although a product might have a higher MoD, its cross-linking efficiency, as reflected by the CrD value, might be relatively low.

## 4. Discussion

In this study, we comprehensively analyzed various commercially available HA fillers using NMR. We focused on crucial parameters such as the MoD, the CrR, and the CrD, which serve as key indicators of structural characteristics affecting the biocompatibility and performance of HA fillers, with the CrD representing a measure of product longevity [14].

The data revealed a broad spectrum of MoD values among the tested fillers, ranging from 17.065% (product 5) to 2.239% (product 7). This variability suggests that clinicians have access to a diverse array of HA fillers, offering unique clinical advantages. Gels with higher MoD values and well-controlled cross-linking will likely offer enhanced structural stability and longer-lasting results when used for medical or cosmetic procedures.

Product 9, with its high CrD and MoD values, may offer enhanced structural stability and endurance. However, while a higher MoD is essential, it is equally important to consider the degree of improperly attached BDDE in conjunction with the CrR. Product 5 had a high MoD, but its CrR value was relatively low (0.086) among the tested fillers. In contrast, product 7 had a low MoD but a high CrR value of 0.222 among the tested fillers (Figure 3). Instances where the MoD is high but the CrR is low indicate inadequate cross-linking, which leads to increased pendant BDDE and reduced structural integrity and cohesiveness (Figure 4).

The findings presented in Table 1 highlight the significant diversity in the CrR values among the tested fillers, ranging from 0.014 (product 8) to 0.394 (product 1). Fillers with high CrR values are expected to demonstrate superior cross-linking efficiencies, resulting in enhanced structural stability and durability. Optimal cross-linking is essential for preventing filler migration and reducing complications, such as lumpiness or unevenness in the treated area. Consequently, clinicians are encouraged to select fillers with high CrR values, particularly for procedures in areas requiring stability and tissue integration.

Similarly, although a high CrR suggests structural stability, a concurrent high MoD indicates significant deviation from the original HA composition, potentially increasing the risk of a foreign substance reaction. Hence, manufacturers should emphasize minimizing the MoD. Products 9 and 13 exhibited similarly elevated CrR levels; nevertheless, the two fillers’ absolute amounts of pendant BDDE impurities differed.

The CrD provides valuable insights into the CrR and MoD, representing the ratio of effective modification; however, the CrD may not determine whether impurities, such as pendant BDDE, are present at high or low concentrations (Figure 3).

Researchers can evaluate the structural integrity and safety of HA gels for clinical use by quantifying cross-linking efficiency. A thorough understanding of the structural attributes of HA gels and the incorporation of pendant BDDE cross-linking can be achieved through various analytical techniques, including mass spectrometry (MS) and liquid chromatography (LC) [15]. NMR provides detailed information about the atomic arrangement and bonding in molecules, allowing for a deeper understanding of the stereochemical properties of cross-linked structures. Furthermore, NMR analysis preserves the integrity of the HA gel structure, eliminating the need to degrade HA into its constituent disaccharides. This preservation is invaluable for discerning structural alterations, modifications, or cross-linking patterns within the gel matrix, which can influence product development. While LC and MS primarily provide information about molecular mass or separated components, they have limitations in detailing structural specifics. NMR has distinct advantages over LC-MS, making it the preferred method for specific structural research and analysis. However, integrating techniques such as MS or LC ensures the comprehensive characterization of HA gel properties.

NMR analysis preserves the integrity of the HA gel structure, eliminating the need to degrade HA into its constituent disaccharides [16]. This preservation is invaluable for discerning structural alterations, modifications, or cross-linking patterns within the gel matrix, influencing product development. NMR provides intricate structural insights; however, integrating techniques such as MS or LC ensures the comprehensive characterization of HA gel properties.

Although NMR analysis offers distinct advantages for studying HA gels, the sample preparation process presents considerable challenges. HA’s inherent gel-like nature makes it unsuitable for direct NMR analysis, necessitating modifications to standard sample preparation protocols. After multiple iterations, we devised a specialized preprocessing method tailored for HA filler analysis. 

A notable deviation from conventional NMR sample preparation is incorporating an enzymatic treatment using chondroitinase ABC. Chondroitinase can selectively remove chondroitin sulfate from samples, leaving only the HA molecules behind. This process ensures that the structural analysis is specific to HA, allowing for a more accurate determination of parameters, such as molecular cross-linking density and MoD. 

The most important modification during the preparation process was the introduction of sample shaking. The extended 48 h incubation period in a shaking incubator was implemented to address the intricate structural complexities inherent to HA filler samples. Notably, whereas the laboratory-manufactured HA gel underwent structural changes when treated with enzymes, the commercially available HA filler retained its gel-like consistency even after 72 h of enzymatic treatment without shaking. The introduction of gentle shaking proved crucial for addressing this unique characteristic.

Although this study offers pivotal insights into HA gel analysis via NMR, certain limitations must be acknowledged. This study investigated the clinical implications of improperly cross-linked BDDE types but did not record direct clinical data or patient outcomes. Therefore, further clinical studies that correlate NMR data with real-world patient responses and safety profiles are needed. 

Implementing rigorous quality control measures and extensive testing protocols is crucial to ensure HA fillers adhere to safety benchmarks, thus minimizing the potential risks linked to residual BDDE. Continued research and clinical investigations are necessary to comprehensively understand the long-term effects and safety issues of pendant BDDE in HA fillers. 

Despite these constraints, our findings provide a basis for using NMR analysis for commercially available HA gels and provide valuable perspectives for product quality control, safety assessments, and clinical performance evaluations. Addressing these limitations in subsequent studies will improve our understanding and practical applications in this domain.

## 5. Conclusions

There is an urgent need for researchers, clinicians, and regulatory entities to assess and monitor the levels of unreacted BDDE in HA fillers and establish stringent quality control measures and expansive testing protocols. Such measures ensure that HA fillers meet established safety benchmarks, thereby reducing the potential hazards associated with residual BDDE. The understanding of the MoD and the CrR, facilitated by NMR analysis, has emerged as an invaluable asset. This analytical approach helps enhance the natural appearance of the fillers and contributes to their biocompatibility, ensuring a comprehensive and positive patient experience in aesthetic interventions.

## Figures and Tables

**Table 1:** Various hyaluronic acid fillers based on key cross-linking parameters.

Product	MoD (%, ^1^H)	MoD (%, ^13^C)	CrR (^13^C)	CrD (%, ^13^C)
1	11.234	8.194	0.394	3.232
2	10.194	9.723	0.085	0.824
3	2.370	2.112	0.117	0.246
4	8.789	7.103	0.143	1.015
5	17.065	12.567	0.086	1.077
6	6.303	4.915	0.118	0.581
7	2.239	1.947	0.227	0.443
8	14.748	9.904	0.014	0.204
9	14.476	12.101	0.333	4.034
10	10.912	9.269	0.1411	1.304
11	4.748	2.999	0.049	0.231
12	8.055	4.747	0.065	0.306
13	7.192	6.147	0.343	2.110

## Data Availability

The original contributions presented in the study are included in the article, further inquiries can be directed to the corresponding author.
